# Phase II study (KAMELEON) of single‐agent T‐DM1 in patients with HER2‐positive advanced urothelial bladder cancer or pancreatic cancer/cholangiocarcinoma

**DOI:** 10.1002/cam4.5893

**Published:** 2023-04-29

**Authors:** Elisabeth G. E. de Vries, Josef Rüschoff, Martijn Lolkema, Josep Tabernero, Luca Gianni, Emile Voest, Derk Jan A. de Groot, Daniel Castellano, Gilles Erb, Julia Naab, Margarita Donica, Regula Deurloo, Michiel S. van der Heijden, Giuseppe Viale

**Affiliations:** ^1^ Department of Medical Oncology University Medical Center Groningen, University of Groningen Groningen The Netherlands; ^2^ Targos Molecular Pathology GmbH Kassel Germany; ^3^ Department of Medical Oncology Erasmus MC Cancer Institute Rotterdam The Netherlands; ^4^ Vall d'Hebron Hospital Campus and Institute of Oncology (VHIO), UVic‐UCC, IOB‐Quiron Barcelona Spain; ^5^ Michelangelo Foundation Milan Italy; ^6^ Netherlands Cancer Institute Amsterdam The Netherlands; ^7^ Oncode Institute Amsterdam The Netherlands; ^8^ Medical Oncology Department Hospital Universitario 12 de Octubre i + 12 Research Institute Madrid Spain; ^9^ F. Hoffmann‐La Roche Ltd. Basel Switzerland; ^10^ IEO, European Institute of Oncology IRCCS Milan Italy; ^11^ University of Milan Milan Italy

**Keywords:** HER2‐positive, KAMELEON, pancreatic cancer, trastuzumab emtansine, urothelial bladder cancer

## Abstract

The antibody‐drug conjugate trastuzumab emtansine (T‐DM1) is approved for human epidermal growth factor receptor 2 (HER2/*ERBB2*)–positive breast cancer. We aimed to study tumor HER2 expression and its effects on T‐DM1 responses in patients with HER2‐positive urothelial bladder cancer (UBC) or pancreatic cancer (PC)/cholangiocarcinoma (CC). In the phase II KAMELEON study (NCT02999672), HER2 status was centrally assessed by immunohistochemistry, with positivity defined as non‐focal homogeneous or heterogeneous overexpression of HER2 in ≥30% of stained cells. We also performed exploratory biomarker analyses (e.g., gene‐protein assay) on tissue samples collected from study participants and consenting patients who failed screening. Of the 284 patients successfully screened for HER2 status (UBC, *n* = 69; PC/CC, *n* = 215), 13 with UBC, four with PC, and three with CC fulfilled eligibility criteria. Due to recruitment difficulty, the sponsor terminated KAMELEON prematurely. Of the five responders in the UBC cohort (overall response rate, 38.5%), HER2 expression was heterogeneous in two and homogeneous in three. The one responder in the PC/CC cohort had PC, and the tumor displayed homogeneous expression. In the biomarker‐evaluable population, composed of screen‐failed and enrolled patients, 24.3% (9/37), 1.5% (1/66), and 8.2% (4/49) of those with UBC, PC, or CC, respectively, had HER2‐positive tumors. In a gene‐protein assay combining in situ hybridization with immunohistochemistry, greater HER2 homogeneity was associated with increased *ERBB2* amplification ratio. In conclusion, KAMELEON showed that some patients with HER2‐positive UBC or PC can respond to T‐DM1 and provided insight into the prevalence of HER2 positivity and expression patterns in three non‐breast tumor types.

## Graphical abstract

The phase II KAMELEON study investigated the efficacy of T‐DM1 in selected HER2‐positive non‐breast tumors. Although KAMELEON was terminated prematurely due to recruitment difficulty, the study demonstrated that patients with HER2‐positive urothelial bladder cancer or pancreatic cancer can respond to T‐DM1. This study provided insight into the prevalence of HER2 positivity and expression patterns in these tumor types and may inform the use of T‐DM1–based regimens in HER2‐positive non‐breast tumors.

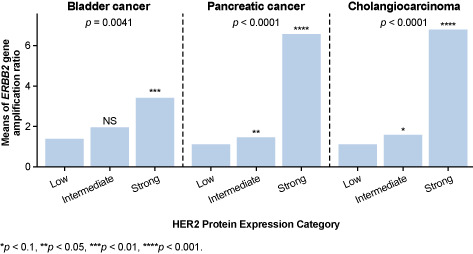

## 1 INTRODUCTION

Gene amplification or overexpression of the protein encoded by the erb‐B2 receptor tyrosine kinase 2 (*ERBB2*) gene, human epidermal growth factor receptor 2 (HER2), is associated with aggressive growth and poor clinical outcomes in a variety of cancers, including breast, ovarian, and gastric.^1^, ^2^ HER2‐targeted therapy improves overall survival (OS) and progression‐free survival (PFS) in patients with advanced HER2‐positive breast or gastric cancer.^3^, ^4^, ^5^, ^6^, ^7^, ^8^ Guidelines recommend the use of immunohistochemistry (IHC) and/or in situ hybridization (ISH) to determine a tumor's HER2 status.^9^, ^10^

By IHC, a breast tumor is considered HER2‐positive if scored as IHC 3+ based on circumferential membrane staining that is complete, intense, and in >10% of tumor cells. By ISH, a breast tumor is considered HER2‐positive if the ratio of *ERBB2* to *chromosome enumeration probe 17* (*CEP17*) is ≥2.0 and the average *ERBB2* copy number is ≥4.0. With equivocal ISH test results (*ERBB2:CEP17* ≥ 2.0 and *ERBB2* copy number <4, as well as ratio *ERBB2:CEP17* < 2.0 and *ERBB2* copy number ≥4), the final decision is made based on the IHC result.^10^ A gastric tumor is considered HER2‐positive if scored as IHC 3+ based on a surgical specimen exhibiting strong complete, basolateral, or lateral membranous reactivity in ≥10% of tumor cells. Also, a gastric biopsy with a cluster of ≥5 cancer cells exhibiting strong complete, basolateral, or lateral membranous reactivity—irrespective of the percentage of cancer cells stained—is considered HER2‐positive. These criteria differ from the breast cancer guideline, which requires circumferential rather than basolateral/lateral membrane staining. HER2 testing guidelines for breast cancer also make no provision for cancer cell clustering. ISH positivity in gastric cancer is similar to that in breast cancer.^9^ However, it has been proposed that HER2 status should no longer be classified simply as negative or positive, but rather as a continuum,^11^ as tumors with low levels of HER2 expression can respond to HER2‐targeted therapy.^12^, ^13^, ^14^

HER2 testing guidelines exist only for tumor types with approved HER2‐targeted therapies (i.e., breast and gastric cancers),^9^, ^10^ but other solid tumors also exhibit perturbations in HER2 protein and *ERBB2* gene levels. For example, the rate of HER2‐positivity in urothelial bladder cancer (UBC) ranges from 17% to 80% based on IHC^15^ and 0%–25% based on ISH.^16^ Additionally, IHC 3+ HER2 overexpression has been reported in 4%–11% of patients with pancreatic cancer.^17^, ^18^ However, there is a discordance between HER2 overexpression and *ERBB2* gene amplification in pancreatic cancer. In one study, only 64% of pancreatic tumors graded as IHC 3+ exhibited *ERBB2* amplification.^18^ HER2 overexpression in pancreatic cancer may be due to gene deregulation rather than amplification, as proposed for intrahepatic cholangiocarcinoma.^18^, ^19^ In a meta‐analysis, estimated HER2 overexpression and *ERBB2* gene amplification rates were higher in extrahepatic biliary tract carcinomas than in intrahepatic cholangiocarcinoma (19.9% vs. 4.8%).^20^ Targeted treatment options are limited for patients with UBC and pancreatic cancer/cholangiocarcinoma. Therefore, HER2‐targeted therapies, like the antibody‐drug conjugate trastuzumab emtansine (T‐DM1), may help address a high unmet need.

In the United States and the European Union, T‐DM1 is approved for use in patients with previously treated HER2‐positive metastatic breast cancer and in patients with HER2‐positive early breast cancer who have residual invasive disease following neoadjuvant treatment. Previous clinical trials have demonstrated the efficacy of HER2‐targeted therapies in non‐breast solid tumors, including biliary tract, non‐small cell lung, colorectal, and bladder cancers,^21^ and several studies have assessed T‐DM1 in non‐breast cancers.^22^, ^23^, ^24^ Although T‐DM1 was not superior to taxane in a study of patients with previously treated, HER2‐positive advanced gastric cancer,^22^ it was an active agent in patients with *ERBB2*‐mutant/HER2‐overexpressing lung cancers^23^, ^24^ and is included as a treatment option for certain patients with non‐breast cancers, such as *ERBB2*‐mutant metastatic non‐small cell lung cancer and HER2‐positive recurrent or metastatic salivary gland tumors.^25^, ^26^ As the efficacy of T‐DM1 in HER2‐positive UBC or pancreatic cancer/cholangiocarcinoma is unknown, KAMELEON sought to assess whether patients with these tumor types would benefit from T‐DM1. However, due to difficulties in enrolling a sufficient number of patients needed within a reasonable time frame, the sponsor terminated KAMELEON prematurely, and the study was unable to address its primary and secondary objectives. Consequently, only a high‐level summary of efficacy, safety, and pharmacokinetic outcomes is provided. Exploratory biomarker analyses conducted on tissue samples from screen‐failed patients and study participants form this report's primary focus. HER2 staining work performed in advance of KAMELEON is provided for completeness.

## 2 MATERIALS AND METHODS

### 2.1 Determination of HER2 thresholds in UBC and pancreatic cancer/cholangiocarcinoma

Although the cutoffs for HER2‐positivity in breast and gastric cancers may be useful in defining similar values in other tumor types, the thresholds for treatment benefit may differ across indications. Thus, in preparation for KAMELEON (ClinicalTrials.gov NCT02999672), pre‐trial biomarker analyses were undertaken to determine a HER2 threshold that would potentially lead to treatment responses in UBC or pancreatic cancer/cholangiocarcinoma. To this end, tumor samples were analyzed centrally using IHC at Targos Molecular Pathology GmbH for HER2 staining intensity (IHC 0, 1+, 2+, or 3+) and pattern (complete vs. incomplete [lateral] membrane staining) via the PATHWAY anti‐HER‐2/neu (4B5) assay (Ventana Medical Systems, Inc.). The PATHWAY anti‐HER‐2/neu [4B5] IHC assay was performed in accordance with the manufacturer's instructions.

### 2.2 Study design and patients

KAMELEON was a multicenter, exploratory, non‐randomized, single‐arm, phase II study (NCT02999672). It was investigator‐initiated, but eventually received corporate sponsorship (Roche). KAMELEON, which was conducted at 10 sites across Italy, Spain, and the Netherlands, sought to determine the best tumor response following treatment with T‐DM1 in patients with HER2‐overexpressing solid tumors. Eligible adults (≥18 years) had HER2‐positive locally advanced (unresectable and not treatable with curative intent) or metastatic UBC or locally advanced or metastatic pancreatic cancer/cholangiocarcinoma measurable per Response Evaluation Criteria in Solid Tumors (RECIST), version 1.1.^27^ HER2 status was determined centrally (Targos Molecular Pathology GmbH) by IHC per the manufacturer's instructions (PATHWAY anti‐HER‐2/neu [4B5] assay, Ventana Medical Systems, Inc.).

In the pre‐trial IHC analyses, 25.0% (11/44) of patients with UBC, 0% (0/62) with pancreatic cancer, and 7.1% (3/42) with cholangiocarcinoma had tumors exhibiting complete membrane staining in ≥30% of stained cells. Since HER2 heterogeneity is prevalent in UBC^28^, ^29^ and the efficacy of T‐DM1 in more heterogeneous cancers is unknown, a decision was made to define HER2 positivity in KAMELEON as tumors exhibiting non‐focal homogeneous or heterogeneous overexpression of HER2 protein (IHC 3+) in ≥30% of tumor cells. Only patients with HER2‐positive UBC, pancreatic cancer, or cholangiocarcinoma were eligible to participate. See Data S1 for additional inclusion/exclusion criteria. Study participants received single‐agent T‐DM1 2.4 mg/kg once weekly (qw) or 3.6 mg/kg every 3 weeks (q3w) until progressive disease, unacceptable toxicity, withdrawal, or the development of an intercurrent illness that in the investigator's opinion would affect patient safety.

This study was performed in compliance with the Declaration of Helsinki, the International Conference on Harmonization Guidelines for Good Clinical Practice, and applicable national and local regulatory requirements. The study protocol was approved by the Independent Ethics Committee or Institutional Review Board at each participating site. Written informed consent was obtained from patients for the clinical and exploratory biomarker analyses.

### 2.3 Outcomes

The primary efficacy objective was to evaluate the best overall response (BOR), with tumor responders defined as individuals with confirmed complete response (CR) or partial response (PR), as determined by the study investigator using RECIST 1.1. Secondary efficacy endpoints included PFS (time from the first day of study treatment until first documented disease progression, as determined by the investigator using RECIST 1.1, or death due to any cause, whichever occurred first) and OS (time from the first day of study treatment until death due to any cause). Adverse events (AEs), which were recorded throughout the study, were coded to the Medical Dictionary for Regulatory Activities (version 21.0) and graded per National Cancer Institute Common Terminology Criteria for Adverse Events (version 4.03). A serious AE was defined as any AE that was fatal or life‐threatening, required or prolonged hospitalization, resulted in persistent or significant disability/incapacity, was a congenital anomaly/birth defect in a neonate/infant born to a mother exposed to study drug, or was a significant medical event (per investigator's opinion). The primary pharmacokinetic objective was to quantify serum/plasma concentrations of T‐DM1 to determine exposure. Additional details regarding assessments are available in the Data S1.

To explore the relationship between biomarkers and tumor response and to evaluate biomarkers that could increase our understanding of the underlying biology of these cancers, the following exploratory biomarker analyses were undertaken: determination of HER2 protein expression pattern in correlation to *ERBB2* copy number by a novel gene‐protein assay combining ISH (INFORM HER2 Dual ISH DNA probe assay, Ventana Medical Systems, Inc.) with IHC (PATHWAY anti‐HER‐2/neu [4B5] assay, Ventana Medical Systems, Inc.) on a single slide. As described previously,^30^, ^31^ the gene‐protein assay was performed on the BenchMark XT platform (Ventana Medical Systems, Inc.), with IHC undertaken prior to ISH. IHC was detected using the iVIEW DAB IHC Detection Kit (Ventana Medical Systems, Inc.) and ISH using a 2,4 dinitrophenyl (DNP)‐labeled *ERBB2* probe and a digoxigenin (DIG)‐labeled *CEN17* probe. *ERBB2* and *CEN17* were detected via the ultraView Silver ISH DNP and ultraView Red ISH DIG Detection Kits, respectively (both Ventana Medical Systems, Inc.), with *ERRB2* developed first. Slides were then counterstained with hematoxylin II (Ventana Medical Systems, Inc.) and visualized via light microscopy, with *ERBB2* appearing as a black signal, *CEN17* as a red signal, and HER2 as a brown stain. Tumors were evaluated in accordance with American Society of Clinical Oncology/College of American Pathologists guidelines for breast cancer.^10^ All assays were performed in accordance with the manufacturer's instructions.

### 2.4 Statistics

The sample size calculation was based on Simon's two‐stage design with early futility analysis to test the null hypothesis (BOR ≤ 5%) against the alternative hypothesis (BOR ≥ 20%). With a one‐sided alpha of 5%, 27 patients were required to reject the null hypothesis with 80% power. To allow for a dropout rate of 10%–15%, 32 patients per cohort were targeted for enrollment. Evidence of potential clinical activity would be concluded if at least four patients in a single cohort responded to study treatment.

The number and percentage of responders (i.e., confirmed CR or PR), with the corresponding two‐sided Clopper–Pearson 90% confidence intervals (CIs), were provided for the primary efficacy endpoint. Time‐to‐event measures were analyzed using the method of Kaplan–Meier; medians and two‐sided 95% CIs were provided when applicable. AEs were summarized using standard frequency tables (number and percentages of patients with events). For patients receiving T‐DM1 2.4 mg/kg qw, blood samples for the pharmacokinetic assessments were drawn during cycle 1 (pre‐dose and 15–30 min post‐dose on Days 1, 8, and 15), cycle 2 (pre‐dose on Day 1), and cycle 4 (pre‐dose and 15–30 min post‐dose on Day 1). For patients receiving T‐DM1 3.6 mg/kg q3w, blood samples were drawn during cycle 1 (pre‐dose and 15–30 min post‐dose on Day 1), cycle 2 (pre‐dose on day 1), and cycle 4 (pre‐dose and 15–30 min post‐dose on Day 1). All efficacy and safety analyses were based on the safety population (patients who received ≥1 dose of T‐DM1). Statistical analyses were performed using SAS version 9.2 or higher. The last patient's last visit occurred April 10, 2018, and the database was locked June 8, 2018.

## 3 RESULTS

### 3.1 Patients

**FIGURE 1:**
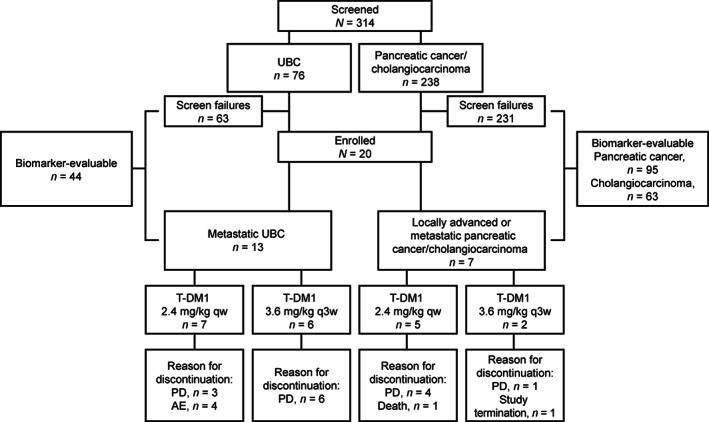
Patient disposition in the KAMELEON study. AE, adverse event; PD, progressive disease; qw, weekly; q3w, every 3 weeks; T‐DM1, trastuzumab emtansine; UBC, urothelial bladder cancer.

KAMELEON was conducted between October 25, 2016, and April 10, 2018. Recruitment was halted on September 22, 2017, due to the difficulty in recruiting enough patients for the primary efficacy analysis within a reasonable timeframe. In the UBC cohort, screening was performed on the primary tumor in ~80% of patients and on metastases in ~20% of patients. In the pancreatic cancer/cholangiocarcinoma cohort, these values were 63% and 37%, respectively. Across all cohorts, ~60% of tissues were derived from resection samples and 40% from biopsies. Among all 314 screened patients, 284 were successfully tested for HER2 overexpression by IHC (69 patients with UBC, 133 with pancreatic cancer, and 82 with cholangiocarcinoma). Of these, 25% (17/69), 4% (5/133), and 7% (6/82) were HER2‐positive, respectively, and 20 patients (UBC, *n* = 13; pancreatic cancer, *n* = 4; cholangiocarcinoma, *n* = 3) met all eligibility criteria (Figure 1). Among the 13 patients enrolled in the UBC cohort, the tumors from eight exhibited heterogeneous HER2 protein expression (30%–70% of tumor cells) by IHC; the other five exhibited homogeneous HER2 staining (>70% of tumor cells). The corresponding values in the pancreatic cancer cohort were three and one. One tumor in the cholangiocarcinoma cohort displayed a heterogeneous expression pattern, and two displayed a homogeneous expression pattern.

All patients with UBC had metastatic disease (Table 1 and Table S1) and discontinued treatment with T‐DM1 due either to progressive disease (PD; 69.2% [9/13]) or an AE (30.8% [4/13]). Among the seven patients in the pancreatic cancer/cholangiocarcinoma cohort, the most common reason for discontinuing T‐DM1 was PD (71.4% [5/7]). The Kaplan–Meier estimated median duration of follow‐up (95% CI) was 7.39 (4.11–10.02) months for the metastatic UBC cohort and 9.23 (1.18–10.87) months for the advanced pancreatic cancer/cholangiocarcinoma cohort, and the median duration of exposure to T‐DM1 (range) was 7.14 (0.1–27.0) weeks and 16.14 (4.1–41.4) weeks, respectively (Table 2).

**TABLE 1:** Baseline demographics and disease characteristics.

	Urothelial bladder cancer (*n* = 13)	Pancreatic cancer/cholangiocarcinoma (*n* = 7)
Median age, years (range)	62.0 (32–78)	62.0 (54–77)
Male, *n* (%)	12 (92.3)	4 (57.1)
Race, *n* (%)		
White	12 (92.3)	7 (100.0)
Unknown	1 (7.7)	0
ECOG PS, *n* (%)		
0	3 (23.1)	5 (71.4)
1	10 (76.9)	2 (28.6)
Primary tumor site, *n* (%)		
Bladder	9 (69.2)	‐
Renal pelvis	3 (23.1)	‐
Ureter	1 (7.7)	‐
Gallbladder	‐	2 (28.6)
Pancreas	‐	4 (57.2)
Bile duct	‐	1 (14.3)
Histology, *n* (%)		
Transitional (urothelial) cell carcinoma	13 (100.0)	‐
Adenocarcinoma	‐	7 (100.0)
Staging at initial diagnosis, *n* (%)		
Pathological	13 (100.0)	‐
Clinical	2 (15.4)	‐
Stage at primary diagnosis, *n* (%)		
II	‐	1 (14.3)
IIB	‐	2 (28.6)
III	‐	1 (14.3)
IV	‐	1 (14.3)
Unknown	‐	2 (28.6)
Disease status at enrollment, *n* (%)		
Locally advanced	0	‐
Metastatic	13 (100.0)	‐
Prior tumor surgery for the cancer of interest, *n* (%)		
No	3 (23.1)	1 (14.3)
Yes	10 (76.9)	6 (85.7)
Prior radiotherapy, *n* (%)		
No	8 (61.5)	6 (85.7)
Yes	5 (38.5)	1 (14.3)
Prior lines of therapy, *n* (%)		
1	2 (15.4)	4 (57.1)
2	5 (38.5)	2 (28.6)
≥3	6 (46.2)	1 (14.3)
Type of prior therapy, *n* (%)		
Chemotherapy	13 (100.0)	7 (100.0)
Immunotherapy	7 (53.8)	0
Prior intravesical therapy, *n* (%)		
No	12 (92.3)	‐
Yes	1 (7.7)	‐

Abbreviations: ECOG, Eastern Cooperative Oncology Group; PS, performance status.

**TABLE 2:** Exposure and efficacy outcomes.

	Urothelial bladder cancer (*n* = 13)	Pancreatic cancer/cholangiocarcinoma (*n* = 7)
Median duration of follow‐up, months (95% CI)^a^	7.39 (4.11–10.02)	9.23 (1.18–10.87)
Median duration of exposure to T‐DM1, weeks (range)	7.14 (0.1–27.0)	16.14 (4.1–41.4)
BOR, *n* (%)		
CR	0	0
PR	5 (38.5)	1 (14.3)
SD	1 (7.7)	3 (42.9)
PD	6 (46.2)	2 (28.6)
NE	1 (7.7)	1 (14.3)
ORR, % (90% CI)^b^	38.5 (16.57, 64.52)	14.3 (0.73, 52.07)
Median DOR, months (95% CI)^a^	3.38 (2.83, 5.52)	—^c^
PFS		
Events, *n* (%)	13 (100.0)	6 (85.7)
Median PFS, months (95% CI)^a^	2.20 (1.18, 4.30)	2.58 (1.31, 9.99)
OS		
Events, *n* (%)	7 (53.8)	1 (14.3)
Median OS, months (95% CI)^a^	7.03 (3.75, NE)	NE (1.45, NE)

Abbreviations: BOR, best overall response; CI, confidence interval; CR, complete response; DOR, duration of response; NE, not evaluable; ORR, overall response rate; OS, overall survival; PD, progressive disease; PFS, progression‐free survival; PR, partial response; SD, stable disease.

^a^ Kaplan–Meier estimate. The corresponding 95% CI was computed using the Brookmeyer and Crowley method.

^b^ The 90% CI was computed using the Clopper–Pearson approach.

^c^ The response duration in the one patient in the pancreatic cancer/cholangiocarcinoma cohort with a PR was 8.6 months.

### 3.2 Efficacy and safety

Five patients in the UBC cohort and one in the pancreatic cancer/cholangiocarcinoma cohort exhibited a PR, corresponding to an overall response rate (ORR; 90% CI) of 38.5% (16.57–64.52%) and 14.3% (0.73–52.07%), respectively. No patient in either cohort had a CR (Table 2). Of the five responding patients with UBC, four received T‐DM1 2.4 mg/kg qw, and one received T‐DM1 3.6 mg/kg q3w (Table S2). In terms of HER2 staining by IHC, the tumors of two of the five responding patients exhibited heterogeneous expression by IHC. One of these heterogeneous tumors showed strong HER2 protein expression and an *ERBB2* amplification ratio by gene‐protein assay of 1.79, and the other exhibited intermediate HER2 protein expression and an *ERBB2* gene amplification ratio by gene‐protein assay of 1.41. Tumor samples from the other three patients exhibited homogeneous HER2 expression by IHC, with two showing strong HER2 protein expression and high *ERBB2* amplification ratios (7.44 and 3.31) and one displaying intermediate HER2 protein expression and an *ERBB2* amplification ratio by gene‐protein assay of 1.65. The one responder in the pancreatic cancer/cholangiocarcinoma cohort had pancreatic cancer and received T‐DM1 2.4 mg/kg qw (Table S2). This patient's tumor displayed homogeneous HER2 protein expression by IHC, strong HER2 protein expression, and a high gene amplification ratio (6.57 by gene‐protein assay).

In total, 84.6% (11/13) of patients in the UBC cohort experienced ≥1 AE, all considered treatment‐related (Table 3). The most common AEs were pyrexia (38.5% [5/13]) and asthenia (30.8% [4/13]). The proportion of patients experiencing grade 3 and grade 4 AEs was 30.8% (*n* = 4) and 0%, respectively. Three patients (23.1%) experienced fatal AEs (pulmonary sepsis, craniocerebral injury, and urinary tract infection; *n* = 1 for each AE); all fatal AEs occurred in patients who received T‐DM1 2.4 mg/kg qw (Table S3). No AE leading to death was considered by the investigator to be related to T‐DM1.

**TABLE 3:** Safety summary.

	Urothelial bladder cancer (*n* = 13)	Pancreatic cancer/cholangiocarcinoma (*n* = 7)
Any AE^a^	11 (84.6)	7 (100.0)
Pyrexia	5 (38.5)	3 (42.9)
Asthenia	4 (30.8)	2 (28.6)
Abdominal pain	0	4 (57.1)
Grade ≥ 3 AE^b^	7 (53.8)	2 (28.6)
Serious AE	6 (46.2)^c^	2 (28.6)^d^
Treatment‐related AE	11 (84.6)	6 (85.7)
AE leading to treatment discontinuation	3 (23.1)^e^	0
AE leading to death	3 (23.1)^e^	0

Abbreviations: AE, adverse event; qw, weekly; T‐DM1, trastuzumab emtansine; UBC, urothelial bladder cancer.

^a^ Only preferred terms reported in ≥4 patients in either cohort are presented.

^b^ No grade ≥3 AE occurred in more than one patient.

^c^ The only serious AE to occur in more than one patient was urinary tract infection, which was reported in two patients. Both patients were administered T‐DM1 2.4 mg/kg qw.

^d^ No serious AE occurred in more than one patient in the pancreatic cancer/cholangiocarcinoma cohort.

^e^ Three of the six serious AEs reported in the UBC cohort led to discontinuation of T‐DM1. These three AEs were also fatal, and each (pulmonary sepsis, craniocerebral injury, and urinary tract infection) occurred in one patient. None was considered by the study investigator to be related to T‐DM1.

Each of the seven patients in the pancreatic cancer/cholangiocarcinoma cohort experienced ≥1 AE; all but one was considered related to T‐DM1 (Table 3 and Table S3). The most common AE among patients with advanced pancreatic cancer/cholangiocarcinoma was abdominal pain (57.1% [4/7]). Grade 3 AEs were reported in two (28.6%) patients. No patient in the pancreatic cancer/cholangiocarcinoma cohort reported a grade 4 or 5 AE.

### 3.3 Pharmacokinetics

T‐DM1 exposures at cycle 1, as measured by maximum concentration (C_max_) and trough concentration (C_min_), following administration of 2.4 mg/kg qw in patients with UBC (*n* = 6–13) were similar to historical data from patients with metastatic breast cancer (*n* = 15) (Figure S1A). Although pharmacokinetic data were limited for patients with pancreatic cancer (*n* = 4–6), a trend of lower exposure was observed for these patients relative to those with either UBC or metastatic breast cancer (Figure S1A). However, T‐DM1 exposures at cycle 1 in patients with pancreatic cancer were comparable to those observed for patients with advanced gastric cancer (*n* = 40–41), in whom a lower T‐DM1 exposure was found relative to patients with metastatic breast cancer.^32^ DM1 C_max_ across cycles was low (2.13–4.01 ng/mL) and largely comparable across indications (Figure S1B; data not shown for C_min_ due to values being below the detection limit).

### 3.4 Gene‐protein assay

Of the 284 patients successfully screened for HER2 overexpression via IHC, 207 consented to the biomarker analyses. However, biomarker data were not available for 55 patients, owing to insufficient or poor‐quality tumor tissue or to assay‐related issues. Thus, the biomarker‐evaluable population was comprised of 152 patients.

**FIGURE 2:**
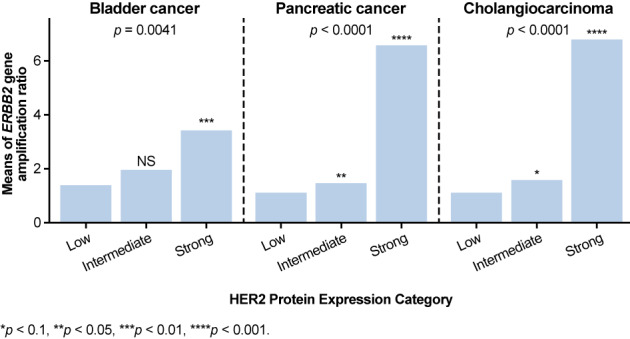
Correlation of HER2 protein expression with the gene amplification ratio by tumor type. Test results from an analysis of variance based on the model: *ERBB2* gene amplification ratio = HER2 protein expression category (low, intermediate, strong) for each indication. Within each indication, two‐by‐two comparisons of HER2 protein expression categories “intermediate” (respectively “strong”) were compared against “low” based on the same method. *ERBB2*, erb‐B2 receptor tyrosine kinase 2; HER2, human epidermal growth factor receptor 2; NS, non‐significant; UBC, urothelial bladder cancer.

In the biomarker‐evaluable population, the tumors of 133, five, seven, and seven patients exhibited no (0% of all stained cells), focal (1%–29% of all stained cells), heterogeneous (30%–70% of all stained cells), and homogeneous (>70% of all stained cells) HER2 protein expression by IHC, respectively. Among patients with UBC, pancreatic cancer, or cholangiocarcinoma in the biomarker‐evaluable population, only 24.3% (9/37), 1.5% (1/66), and 8.2% (4/49), respectively, exhibited heterogeneous or homogeneous expression of HER2 (Table 4). The pattern of HER2 staining by indication is summarized in Table 4. The tumors of 121, 20, and 11 patients exhibited low, intermediate, and strong IHC staining, respectively (Table 4). For each of the three tumor types, the data suggest a positive association between HER2 protein expression level and *ERBB2* amplification ratio (Figure 2 and Figure S2). Similar results were found when examining the *ERBB2* gene amplification ratio in different HER2 staining intensity regions (i.e., low, intermediate, strong) (Figure S3). In the gene‐protein assay, increasing homogeneity at the protein level was associated with increases in the *ERBB2* amplification ratio (Figure S4).

**TABLE 4:** HER2 gene‐protein results by indication.

	Patients eligible for exploratory biomarker analysis			
	UBC	Pancreatic cancer	Cholangiocarcinoma	Total
	(*n* = 44)	(*n* = 95)	(*n* = 63)	(*N* = 202)^a^
Heterogeneity of HER2 protein expression (percentage of positively stained tumor cells), *n* (%)				
Negative (0%)	24 (54.5)	64 (67.4)	45 (71.4)	133 (65.8)
Focal (1%–29%)	4 (9.1)	1 (1.1)	0	5 (2.5)
Heterogeneous (30%–70%)	4 (9.1)	0	3 (4.8)	7 (3.5)
Homogeneous (>70%)	5 (11.4)	1 (1.1)	1 (1.6)	7 (3.5)
Missing	7 (15.9)	29 (30.5)	14 (22.2)	50 (24.8)
HER2 protein expression level, *n* (%)				
Low	20 (45.5)	59 (62.1)	42 (66.7)	121 (59.9)
Intermediate	9 (20.5)	6 (6.3)	5 (7.9)	20 (9.9)
Strong	8 (18.2)	1 (1.1)	2 (3.2)	11 (5.4)
Missing	7 (15.9)	29 (30.5)	14 (22.2)	50 (24.8)

	Biomarker‐evaluable population			
	UBC	Pancreatic cancer	Cholangiocarcinoma	Total
	(*n* = 37)	(*n* = 66)	(*n* = 49)	(*N* = 152)
Median *ERBB2* gene amplification ratio (range)	1.340 (0.76–7.44)	1.035 (0.78–6.57)	1.080 (0.50–12.12)	1.100 (0.50–12.12)

*Note*: For UBC, only complete staining was considered. For pancreatic cancer and cholangiocarcinoma, complete and incomplete staining were considered.

Abbreviations: *ERBB2*, erb‐B2 receptor tyrosine kinase 2; HER2, human epidermal growth factor receptor 2; UBC, urothelial bladder cancer.

^a^ A total of 207 patients consented to exploratory gene‐protein assay analysis; however, 5 samples could not be tested due to tissue wash off during the staining procedure or lack of appropriate material (no tumor content or insufficient tumor content on the slide).

## 4 DISCUSSION

In this phase II study of non‐breast tumor types, HER2‐positivity was detected via gene‐protein assay in 24.3% of patients with UBC, 1.5% with pancreatic cancer, and 8.2% with cholangiocarcinoma. The relatively low prevalence of HER2‐positivity resulted in poor accrual and premature termination of the study. Although centralized HER2 testing may help identify these individuals, the recruitment of such patients is likely to be prohibitively slow if HER2‐positivity rates are as low as those encountered, especially in pancreatic cancer. For biomarker‐driven clinical studies to succeed, we recommend that HER2 testing become part of the standard diagnostic work‐up of non‐breast cancers. Moreover, novel antibody‐drug conjugates like trastuzumab deruxtecan and trastuzumab duocarmazine have resulted in clinically meaningful responses in patients with tumors expressing low HER2 levels,^12^, ^13^, ^14^ indicating that the thresholds for HER2‐positivity must also be evaluated in the context of an evolving treatment landscape.

For each of the three cancers studied in KAMELEON, no meaningful differences were observed regarding the proportion of patients with IHC 3+ tumors exhibiting heterogeneous vs. homogenous HER2 staining. Among patients with metastatic UBC, the HER2 staining pattern did not appear to influence the response to T‐DM1, but the number of responders was too small to make a reliable comparison (*n* = 5). HER2 heterogeneity (30%–79% of stained cells) has been reported in ~10% of advanced breast tumors^33^ and 30% of advanced gastric tumors.^34^ HER2 heterogeneity has been associated with shorter disease‐free survival and PFS and reduced pathologic CR rates in patients with HER2‐positive breast cancer after HER2‐targeted treatment.^30^, ^33^, ^35^, ^36^, ^37^ However, the data are conflicting for HER2‐positive gastric cancer. Some researchers have observed less favorable clinical outcomes in those with heterogeneous vs. homogeneous HER2 expression,^38^, ^39^ while others have observed the converse.^40^, ^41^ The phase III ToGA study compared trastuzumab plus chemotherapy vs. chemotherapy alone in patients with HER2‐positive advanced gastric or gastroesophageal junction tumors. Patients with IHC 2+ and IHC 3+ scores showed an OS benefit with trastuzumab plus chemotherapy vs. chemotherapy alone, regardless of variability in HER2 staining, with a numerically greater benefit in patients with less variable HER2 protein expression (>30% of stained cells).^42^

The concordance between HER2 IHC score and the gene‐protein assay and between ISH and the gene‐protein assay has been estimated at 93% and 96%, respectively.^43^ In the gene‐protein assay, HER2 protein expression was positively associated with *ERBB2* amplification in IHC 3+ tumors, with a higher *ERBB2* amplification ratio observed in tumors exhibiting homogeneous vs. focal or heterogeneous HER2 staining. In terms of HER2 expression level, eight patients had UBC tumors categorized as strong (a reasonable number of samples for performing a correlation analysis). Only one patient with pancreatic cancer and two with cholangiocarcinoma had tumors categorized as strong. In patients with IHC 1–2+ tumors, a positive association with *ERBB2* copy number was seen in UBC, but the relationship was less evident in pancreatic cancer and cholangiocarcinoma. Although intriguing, the results of the biomarker analyses should be interpreted with caution as they were exploratory. In advanced breast cancer, HER2 heterogeneity has been associated with the presence of certain mutations, such as in *PIK3CA*,^33^ but it is unknown whether HER2‐positive UBC, pancreatic, or cholangiocarcinoma tumors exhibiting focal or heterogeneous HER2 expression harbor‐specific mutational drivers.

In contrast to guidelines developed for breast and gastric cancers that also consider *ERBB2* copy number,^9^, ^10^ HER2‐positivity in KAMELEON was based solely on protein expression. This is a potential limitation, particularly if there is discordance between *ERBB2* copy number and HER2 overexpression in UBC, pancreatic cancer, or cholangiocarcinoma. In HER2‐positive breast cancer, the concordance between HER2 at the protein and genetic levels is high. For example, in an Australian study examining 53,402 ISH tests, 196 were identified as discordant to IHC performed at the local laboratory. Upon ISH re‐testing at a central laboratory, 12 (6.1%) remained discordant, with only one sample found to be a false‐positive (IHC 3+/ISH‐negative). The other 11 samples were considered either IHC 0 or 1+ (per local and central testing) and displayed low *ERBB2* amplification; one also exhibited clonal heterogeneity.^44^ To improve clarity, the definition of IHC 2+ has been revised by the American Society of Clinical Oncology/College of American Pathologists.^10^ In HER2‐positive gastric cancer, *ERBB2* copy number predicts the response to trastuzumab plus chemotherapy,^45^ but we were unable to assess whether *ERBB2* copy number affected the observed responses to T‐DM1. Furthermore, our study did not distinguish HER2 testing results from primary versus metastatic tumor tissue. Prior studies have noted discordance in HER2 status between the primary tumor and metastases in multiple cancer types, including breast, endometrial, and urothelial.^46^, ^47^, ^48^, ^49^, ^50^, ^51^ It is therefore possible that the HER2 homogeneity/heterogeneity observed in the tested tumors may not reflect HER2 status at other tumor sites, including treatment‐responsive tumors. In addition to these caveats and the exploratory nature of the biomarker analyses, other limitations of KAMELEON include its non‐randomized design and small sample size.

Due to early termination, KAMELEON was unable to address its primary and secondary objectives fully. Per the original study design, an objective response (CR or PR) in at least four of 27 patients per cohort would have been regarded as evidence of promising efficacy. Five of 13 patients with previously treated HER2‐positive metastatic UBC‐administered single‐agent T‐DM1 exhibited a PR, resulting in an ORR of 38.5%. However, the treatment of metastatic UBC has evolved since KAMELEON was initially planned. PD‐1 or PD‐L1 checkpoint inhibitors (pembrolizumab or atezolizumab), enfortumab vedotin, and erdafitinib were granted accelerated approval by the United States Food and Drug Administration for this indication. For the pancreatic cancer/cholangiocarcinoma cohort, it was not possible to conclude the presence/absence of an efficacy signal due to the small number of enrolled patients (*n* = 7). Of note, the AEs observed in this study were generally consistent with the known safety profile of T‐DM1. Importantly, the three AEs leading to death were not judged by study investigators to be treatment‐related.

Several early‐phase studies exploring HER2‐targeted therapies in non‐breast tumors, as well as a T‐DM1 combination therapy in breast cancer, may have implications for the treatment of the tumor types studied in KAMELEON. In a phase I study, trastuzumab deruxtecan led to an almost 80% reduction in tumor size in a patient with cholangiocarcinoma.^52^ These data suggest that there may be promise in targeting HER2 in non‐breast/non‐gastric tumor types. To this end, a phase II study (NCT02675829) of T‐DM1 in patients with *ERBB2*‐amplified or *ERBB2*‐mutated cancers—irrespective of tumor histology—is underway. There may also be utility in combining T‐DM1 with other therapies, such as immune checkpoint inhibitors. Phase 3 studies of T‐DM1 plus atezolizumab (NCT04740918, NCT04873362) are currently recruiting. Results from these trials may ultimately help inform on the use of T‐DM1‐based regimens in HER2‐positive non‐breast tumors, such as those studied in KAMELEON.

In conclusion, KAMELEON provided preliminary insight into the prevalence of HER2‐positivity, as well as the patterns of HER2 expression, in patients with locally advanced or metastatic non‐breast tumors. The efficacy of single‐agent T‐DM1 observed in patients with advanced UBC is encouraging and suggests that HER2 may represent a viable treatment target for these patients. This is supported by data from a phase I study of trastuzumab duocarmazine, where four of 16 patients with HER2‐expressing urothelial cancer achieved PR.^12^ Data were too limited in KAMELEON to draw conclusions regarding the efficacy of HER2‐targeted therapy in advanced pancreatic cancer or cholangiocarcinoma, but one patient with pancreatic cancer experienced PR following treatment with T‐DM1.

## AUTHOR CONTRIBUTIONS

**Daniel Castellano:** Formal analysis (equal); writing – review and editing (equal). **Derk Jan A. de Groot:** Conceptualization (equal); methodology (equal); writing – review and editing (equal). **Elisabeth de Vries:** Conceptualization (equal); formal analysis (equal); investigation (equal); methodology (equal); resources (equal); writing – original draft (equal); writing – review and editing (equal). **Emile E. Voest:** Conceptualization (equal); investigation (equal); methodology (equal); resources (equal); writing – review and editing (equal). **Gilles Erb:** Conceptualization (equal); resources (equal); writing – review and editing (equal). **Giuseppe Viale:** Conceptualization (equal); formal analysis (equal); methodology (equal); validation (equal); writing – review and editing (equal). **Josep Tabernero:** Conceptualization (equal); formal analysis (equal); methodology (equal); writing – review and editing (equal). **Julia Naab:** Data curation (equal); resources (equal); writing – review and editing (equal). **Luca Gianni:** Conceptualization (equal); investigation (equal); methodology (equal); resources (equal); writing – review and editing (equal). **Martijn Lolkema:** Conceptualization (equal); investigation (equal); methodology (equal); resources (equal); writing – original draft (equal); writing – review and editing (equal). **Margarita Donica:** Formal analysis (equal); writing – review and editing (equal). **M. S. van der Heijden:** Investigation (equal); resources (equal); writing – review and editing (equal). **Regula Deurloo:** Conceptualization (equal); formal analysis (equal); investigation (equal); methodology (equal); resources (equal); supervision (equal); writing – review and editing (equal). **Josef Rüschoff:** Conceptualization (equal); formal analysis (equal); methodology (equal); validation (equal); writing – review and editing (equal).

## CONFLICT OF INTEREST STATEMENT

EGEdV reports institutional financial support for serving as an advisor to Daiichi Sankyo, Merck, National Surgical Adjuvant Breast and Bowel Project, Pfizer, Sanofi, and Synthon, as well as institutional financial support for participating in clinical trials or undertaking contracted research for Amgen, AstraZeneca, Bayer, Chugai Pharma, CytomX Therapeutics, G1 Therapeutics, Genentech, Nordic Nanovector, Radius Health, Regeneron, Roche, Crescendo, Servier, and Synthon. JR reports institutional (Targos) financial support for biomarker testing, has served as advisor to Roche, Genentech, Merck Sharpe & Dohme, Daiichi Sankyo, AstraZeneca, GlaxoSmithKline, and Exact Sciences, and is co‐founder of Targos. ML has received research grants to the institute from Sanofi, JnJ, MSD, and Astellas, has served as advisor to Incyte, Amgen, Janssen Cilag B.V., Bayer, Servier, Roche, INCa, Pfizer, Sanofi Aventis Netherlands BV, Astellas, Astra Zeneca, Merck Sharp and Dome, Novartis, and Julius Clinical. JT reports personal financial interest in the form of scientific consultancy role for Array Biopharma, AstraZeneca, Avvinity, Bayer, Boehringer Ingelheim, Chugai, Daiichi Sankyo, F. Hoffmann‐La Roche Ltd, Genentech Inc, HalioDX SAS, Hutchison MediPharma International, Ikena Oncology, IQVIA, Lilly, Menarini, Merck Serono, Merus, MSD, Mirati, Neophore, Novartis, Orion Biotechnology, Peptomyc, Pfizer, Pierre Fabre, Samsung Bioepis, Sanofi, Seattle Genetics, Scandion Oncology, Servier, Taiho, Tessa Therapeutics, and TheraMyc.; reports educational collaboration with Imedex, Medscape Education, MJH Life Sciences, PeerView Institute for Medical Education, and Physicians Education Resource (PER); and declares institutional financial interest in the form of financial support for clinical trials or contracted research for Amgen Inc, Array Biopharma Inc, AstraZeneca Pharmaceuticals LP, BeiGene, Boehringer Ingelheim, Bristol‐Myers Squibb, Celgene, Debiopharm International SA, F. Hoffmann‐La Roche Ltd, Genentech Inc, HalioDX SAS, Hutchison MediPharma International, Janssen‐Cilag SA, MedImmune, Menarini, Merck Health KGAA, Merck Sharp & Dohme, Merus NV, Mirati, Novartis Farmacéutica SA, Pfizer, Pharma Mar, Sanofi Aventis Recherche & Développement, Servier, Taiho Pharma USA Inc, Spanish Association Against Cancer Scientific Foundation, and Cancer Research UK. LG has served on advisory boards for ADC Therapeutics, AstraZeneca, Celgene, Eli Lilly, G1 Therapeutics, Genentech, Genomic Health, Merck Sharp & Dohme, Oncolytics Biotech, Odonate Therapeutics, Onkaido Therapeutics, Roche, Pfizer, Taiho Pharmaceutical, Hexal Sandoz, Seattle Genetics, Synthon, Zymeworks, and Sanofi‐Aventis; has served as a consultant for selected programs of Forty Seven (CD47), GENENTA, METIS Precision Medicine, Novartis, Odonate Therapeutics, Revolution Medicines, Synaffix, Zymeworks, Menarini Ricerche, Amgen, and Biomedical Insights Inc; has received research support paid to his institution from Zymeworks and Revolution Medicines; and is co‐inventor of European Patent Application N. 12195182.6 and 12196177.5, titled “PDL‐1 expression in anti‐HER2 therapy”‐Roche (no compensation provided). EV has served as a consultant for Roche with an honorarium given to his institution, and his institution received research funding from Roche. DJAdG has no disclosures to report. DC has served as a consultant or advisor to Janssen Oncology, Roche/Genentech, Astellas Pharma, AstraZeneca, Pfizer, Novartis, Ipsen, Bristol‐Myers Squibb, MSD Oncology, Bayer, Lilly, Sanofi, Pierre Fabre, and Boehringer Ingelheim; has received research funding from Janssen Oncology; has received support for travel/accommodations from Pfizer, Roche, Bristol‐Myers Squibb, and AstraZeneca Spain. GE is a salaried employee of Roche. JN is an employee of Hays AG contracted by Roche for technical and operational support of tissue sample analysis and data management in the conduct of the study. MD was an employee at Roche at the time work was conducted. RD is a salaried employee of, and owns stock in, Roche. MSvdH has received research grants (paid to the institute) from BMS, AstraZeneca, Roche and 4SC and has served as an advisor (fees paid to the institute) to Roche, Pfizer, Astellas, Astra Zeneca, Merck Sharp and Dome, BMS, and Janssen. GV has served as a consultant to Novartis, Roche, Merck Sharpe & Dohme, Bayer, Menarini, Daiichi Sankyo, and AstraZeneca.

## Supporting information


Data S1.

## Data Availability

Qualified researchers may request access to de‐identified patient‐level data through the Clinical Study Data Request platform (www.clinicalstudydatarequest.com) and will be provided with accompanying clinical study documentation (protocol and any associated amendments, annotated case report form, reporting and analysis plan, dataset specifications, clinical study report). Researchers requesting access to clinical study documentation only can do so via the following link: http://www.roche.com/research_and_development/who_we_are_how_we_work/clinical_trials/our_commitment_to_data_sharing/clinical_study_documents_request_form.htm. Documents are made available on application, per scope and timing criteria as published on the Clinical Study Data Request platform.
