# Supplementary material for: Phase II study (KAMELEON) of single‐agent T‐DM1 in patients with HER2‐positive advanced urothelial bladder cancer or pancreatic cancer/cholangiocarcinoma
Source: Cancer Med. 2023 Apr 29;12(11):12071–83. doi: 10.1002/cam4.5893 (PMC10278525; doi:10.1002/cam4.5893)
Supplement: Supplementary file 1 — Data S1. [file CAM4-12-12071-s001.pdf]

ONLINE SUPPLEMENT

METHODS

Inclusion/exclusion criteria

Participants must have previously received ≥1 anti-cancer treatment (platinum-based for patients with urothelial bladder cancer [UBC]) and exhausted all other standard treatment options, as well as had an Eastern Cooperative Oncology Group performance status score of 0–2, adequate organ function, no significant cardiac history, and a left ventricular ejection fraction of ≥50%. Exclusion criteria included prior irradiation of the target lesion(s) or treatment with a human epidermal growth factor receptor 2 (HER2)–targeted agent; focal HER2 expression (immunohistochemistry 3+ in <30% of tumor cells); brain metastasis as the sole site of metastatic disease and/or the presence of symptomatic or uncontrolled brain metastasis; uncontrolled hypertension (systolic blood pressure >150 mmHg and/or diastolic blood pressure >100 mmHg); severe, uncontrolled systemic disease; unstable angina pectoris; history of symptomatic congestive heart failure per any New York Heart Association criteria or ventricular arrhythmia requiring treatment; history of myocardial infarction in the 6 months prior to screening; history of another malignancy in the 5 years prior to screening (except for appropriately treated carcinoma *in situ* of the cervix, non-melanoma skin carcinoma, stage I uterine cancer, or other cancer with an outcome similar to the aforementioned); clinically significant bleeding in the 30 days prior to enrollment; and major surgery or significant

traumatic injury in the 28 days prior to randomization or an expected need for major surgery during study treatment.

Study design

As part of a safety run-in, six patients in each cohort (UBC or pancreatic cancer/cholangiocarcinoma) received intravenous infusions of trastuzumab emtansine (T-DM1) 2.4 mg/kg weekly (qw) and were assessed on an ongoing basis for tolerability (as defined in the independent Data Review Committee [iDMC] charter). Recruitment to each cohort was suspended until all six patients had completed the second treatment cycle; as each treatment cycle was of 21 days' duration, this was equivalent to week 6. Based on the available tolerability and safety data, the iDMC was to determine whether an additional 26 patients per cohort were to be recruited and administered T-DM1 2.4 mg/kg qw or if an additional 32 patients per cohort were to be recruited and administered T-DM1 3.6 mg/kg every 3 weeks (q3w), the recommended regimen for the treatment of HER2-positive breast cancer. If the iDMC had decided that treatment with T-DM1 3.6 mg/kg q3w would be activated, then the six patients originally administered T-DM1 2.4 mg/kg qw were allowed to switch if additional clinical benefit was expected. However, if patients had responded to T-DM1 2.4 mg/kg qw, then it was recommended that they continue with that regimen. If any of the original six patients in either cohort experienced progressive disease, no regimen switch was allowed, and the patient was discontinued from the study.

Assessments and statistics

Tumor responses were assessed every 6 weeks per Response Evaluation Criteria in Solid Tumors, version 1.1.[1] Patients without a post-baseline tumor assessment were considered non-responders. Best overall response (BOR) was defined as the best response recorded from the first day of study treatment until disease progression/recurrence or death. To be considered a responder (complete response [CR] or partial response [PR]), changes in tumor measurements must have been confirmed by repeat assessments performed no less than 4 weeks after the response criteria were first met (i.e., patients needed to have two consecutive assessments of CR or PR to be regarded as responders).

A Simon's two-stage design [2] was implemented to permit early termination of the study if there was no evidence of efficacy. In the first stage, 13 patients per cohort were to be accrued, with BOR evaluated 12 weeks after the thirteenth patient had been enrolled. If no objective response (CR or PR) was observed among these 13 patients, the study would be stopped for the respective cohort. Otherwise, the second stage would be activated, and 14 additional patients (27 patients in total) per cohort would be recruited. Recruitment could have been suspended after the thirteenth patient in a cohort had been enrolled if no responses were observed in the previous 12 patients. If at least four of 27 patients exhibited a response, then the null hypothesis (BOR ≤5%) was to be rejected, and the alternative hypothesis (BOR ≥20%) was to be accepted. If there were ≤3 responders, then the null hypothesis was to be accepted. To allow for a dropout rate of 10–15% and to ensure that ≥27 patients would have efficacy data available, 32 patients per cohort were targeted for enrollment.

Progression-free survival (PFS) was defined as the time from the first dose of T-DM1 to investigator-assessed disease progression or death from any cause. Overall survival (OS) was defined as the time from the first dose of T-DM1 to death from any cause. The number and percentage of responders, with the corresponding Clopper-Pearson 90% confidence intervals (CIs), were calculated. PFS and OS (and associated 95% CIs) were estimated using the Kaplan–Meier approach.

Adverse event (AE) grading was based on general guidelines from National Cancer Institute Common Terminology Criteria for Adverse Events (version 4.03). Grade 1 denoted asymptomatic or mild symptoms, with no intervention indicated. Grade 2 denoted moderate symptoms with minimal, local, or noninvasive intervention indicated. Grade 3 denoted severe or medically significant but not immediately life-threatening symptoms, with hospitalization or prolongation of hospitalization indicated. Grade 4 denoted life-threatening consequences, with urgent intervention indicated. Grade 5 denoted death due to an AE.

REFERENCES

1. Eisenhauer EA, Therasse P, Bogaerts J, Schwartz LH, Sargent D, Ford R, Dancey J, Arbuck S, Gwyther S, Mooney M, Rubinstein L, Shankar L, Dodd L, Kaplan R, Lacombe D, Verweij J. New response evaluation criteria in solid tumours: revised RECIST guideline (version 1.1). *Eur J Cancer* 2009; 45(2): 228–247.
2. Simon R. Optimal two-stage designs for phase II clinical trials. *Control Clin Trials* 1989; 10(1): 1–10.

Supplemental Table 1. Baseline demographics and disease characteristics by dose

	**Urothelial bladder cancer**			**Pancreatic cancer/cholangiocarcinoma**		
	**T-DM1 2.4 mg/kg qw (*n* = 7)**	**T-DM1 3.6 mg/kg q3w (*n* = 6)**	**Total (*n* = 13)**	**T-DM1 2.4 mg/kg qw (*n* = 5)**	**T-DM1 3.6 mg/kg q3w (*n* = 2)**	**Total (*n* = 7)**
Median age, years (range)	50.0 (38–78)	66.0 (32–69)	62.0 (32–78)	62.0 (54–65)	69.5 (62–77)	62.0 (54–77)
Male, *n* (%)	6 (85.7)	6 (100.0)	12 (92.3)	3 (60.0)	1 (50.0)	4 (57.1)
Race, *n* (%)						
White	6 (85.7)	6 (100.0)	12 (92.3)	5 (100.0)	2 (100.0)	7 (100.0)
Unknown	1 (14.3)	0	1 (7.7)	0	0	0
ECOG PS, *n* (%)						
0	1 (14.3)	2 (33.3)	3 (23.1)	3 (60.0)	2 (100.0)	5 (71.4)
1	6 (85.7)	4 (66.7)	10 (76.9)	2 (40.0)	0	2 (28.6)
Primary tumor site, *n* (%)						
Bladder	4 (57.1)	5 (83.3)	9 (69.2)	-	-	-
Renal pelvis	2 (28.6)	1 (16.7)	3 (23.1)	-	-	-
Ureter	1 (14.3)	0	1 (7.7)	-	-	-
Gallbladder	-	-	-	2 (40.0)	0	2 (28.6)
Pancreas	-	-	-	2 (40.0)	2 (100.0)	4 (57.2)
Bile duct	-	-	-	1 (20.0)	0	1 (14.3)
Histology, *n* (%)						
Transitional (urothelial) cell carcinoma	7 (100.0)	6 (100.0)	13 (100.0)	-	-	-
Adenocarcinoma	-	-	-	5 (100.0)	2 (100.0)	7 (100.0)
Staging at initial diagnosis, *n* (%)						
Pathological	7 (100.0)	6 (100.0)	13 (100.0)	-	-	-
Clinical	2 (28.6)	0	2 (15.4)	-	-	-
Stage at primary diagnosis, *n* (%)						
II	-	-	-	1 (20.0)	0	1 (14.3)
IIB	-	-	-	2 (40.0)	0	2 (28.6)
III	-	-	-	0	1 (50.0)	1 (14.3)

IV<br>Unknown	-<br>-	-<br>-	-<br>-	1 (20.0)<br>1 (20.0)	0<br>1 (50.0)	1 (14.3)<br>2 (28.6)
Disease status at enrollment, *n* (%)						
Locally advanced	0	0	0	-	-	-
Metastatic	7 (100.0)	6 (100.0)	13 (100.0)	-	-	-
Prior tumor surgery for the cancer of interest, *n* (%)						
No	1 (14.3)	2 (33.3)	3 (23.1)	1 (20.0)	0	1 (14.3)
Yes	6 (85.7)	4 (66.7)	10 (76.9)	4 (80.0)	2 (100.0)	6 (85.7)
Prior radiotherapy, *n* (%)						
No	4 (57.1)	4 (66.7)	8 (61.5)	5 (100.0)	1 (50.0)	6 (85.7)
Yes	3 (42.9)	2 (33.3)	5 (38.5)	0	1 (50.0)	1 (14.3)
Prior lines of therapy, *n* (%)						
1	1 (14.3)	1 (16.7)	2 (15.4)	2 (40.0)	2 (100.0)	4 (57.1)
2	3 (42.9)	2 (33.3)	5 (38.5)	2 (40.0)	0	2 (28.6)
≥3	3 (42.9)	3 (50.0)	6 (46.2)	1 (20.0)	0	1 (14.3)
Type of prior therapy, *n* (%)						
Chemotherapy	7 (100.0)	6 (100.0)	13 (100.0)	5 (100.0)	2 (100.0)	7 (100.0)
Immunotherapy	3 (42.9)	4 (66.7)	7 (53.8)	0	0	0
Prior intravesical therapy, *n* (%)						
No	6 (85.7)	6 (100.0)	12 (92.3)	-	-	-
Yes	1 (14.3)	0	1 (7.7)	-	-	-

ECOG, Eastern Cooperative Oncology Group; PS, performance status; qw, weekly; q3w, every 3 weeks; T-DM1, trastuzumab emtansine; UBC, urothelial bladder cancer.

Supplemental Table 2. Efficacy outcomes by dose

	Urothelial bladder cancer			Pancreatic cancer/cholangiocarcinoma		
	**T-DM1 2.4 mg/kg qw (*n* = 7)**	**T-DM1 3.6 mg/kg q3w (*n* = 6)**	**Total (*n* = 13)**	**T-DM1 2.4 mg/kg qw (*n* = 5)**	**T-DM1 3.6 mg/kg q3w (*n* = 2)**	**Total (*n* = 7)**
BOR, *n* (%)						
CR	0	0	0	0	0	0
PR	4 (57.1)	1 (16.7)	5 (38.5)	1 (20.0)	0	1 (14.3)
SD	1 (14.3)	0	1 (7.7)	1 (20.0)	2 (100.0)	3 (42.9)
PD	1 (14.3)	5 (83.3)	6 (46.2)	2 (40.0)	0	2 (28.6)
NE	1 (14.3)	0	1 (7.7)	1 (20.0)	0	1 (14.3)
ORR, % (90% CI)[a]	57.1 (22.53, 87.12)	16.7 (0.85, 58.18)	38.5 (16.57, 64.52)	20.0 (1.02, 65.74)	0 (0.00, 77.64)	14.3 (0.73, 52.07)
Median DOR, months (95% CI)[b]	3.14 (2.83, 3.91)	5.52 (NE, NE)	3.38 (2.83, 5.52)	-[c]	-	-[c]
PFS						
Events, *n* (%)	-	-	13 (100.0)	-	-	6 (85.7)
Median PFS, months (95% CI)[b]	-	-	2.20 (1.18, 4.30)	-	-	2.58 (1.31, 9.99)
OS						
Events, *n* (%)	-	-	7 (53.8)	-	-	1 (14.3)
Median OS, months (95% CI)[b]	-	-	7.03 (3.75, NE)	-	-	NE (1.45, NE)

BOR, best overall response; CI, confidence interval; CR, complete response; DOR, duration of response; NE, not evaluable; ORR, overall response rate; OS, overall survival; PD, progressive disease; PFS, progression-free survival; PR, partial response; SD, stable disease; qw, weekly; q3w, every 3 weeks; T-DM1, trastuzumab emtansine; UBC, urothelial bladder cancer.

[a]The 90% CI was computed using the Clopper–Pearson approach.

[b]Kaplan–Meier estimate. The corresponding 95% CI was computed using the Brookmeyer and Crowley method.

[c]The response duration in the one patient in the pancreatic cancer/cholangiocarcinoma cohort with a PR was 8.6 months.

Supplemental Table 3. Safety summary by dose

	**Urothelial bladder cancer**			**Pancreatic cancer/cholangiocarcinoma**		
	**T-DM1 2.4 mg/kg qw (*n* = 7)**	**T-DM1 3.6 mg/kg q3w (*n* = 6)**	**Total (*n* = 13)**	**T-DM1 2.4 mg/kg qw (*n* = 5)**	**T-DM1 3.6 mg/kg q3w (*n* = 2)**	**Total (*n* = 7)**
Any AE[a]	7 (100.0)	4 (66.7)	11 (84.6)	5 (100.0)	2 (100.0)	7 (100.0)
Pyrexia	4 (57.1)	1 (16.7)	5 (38.5)	3 (60.0)	0	3 (42.9)
Asthenia	3 (42.9)	1 (16.7)	4 (30.8)	2 (40.0)	0	2 (28.6)
Abdominal pain	0	0	0	3 (60.0)	1 (50.0)	4 (57.1)
Grade ≥3 AE[b]	6 (85.7)	1 (16.7)	7 (53.8)	2 (40.0)	0	2 (28.6)
Serious AE	5 (71.4)	1 (16.7)	6 (46.2)[c]	2 (40.0)	0	2 (28.6)[d]
Treatment-related AE	7 (100.0)	4 (66.7)	11 (84.6)	4 (80.0)	2 (100.0)	6 (85.7)
AE leading to treatment discontinuation	3 (42.9)[e]	0	3 (23.1)[e]	0	0	0
AE leading to death	3 (42.9)[e]	0	3 (23.1)[e]	0	0	0

AE, adverse event; qw, weekly; q3w, every 3 weeks; T-DM1, trastuzumab emtansine; UBC, urothelial bladder cancer.

[a]Only preferred terms reported in ≥4 patients in either cohort are presented.

[b]No grade ≥3 AE occurred in more than one patient.

[c]The only serious AE to occur in more than one patient was urinary tract infection, which was reported in two patients. Both patients were administered T-DM1 2.4 mg/kg qw.

[d]No serious AE occurred in more than one patient in the pancreatic cancer/cholangiocarcinoma cohort.

[e]Three of the six serious AEs reported in the UBC cohort led to discontinuation of T-DM1. These three AEs were also fatal, and each (pulmonary sepsis, craniocerebral injury, and urinary tract infection) occurred in one patient. None was considered by the study investigator to be related to T-DM1.

(A)

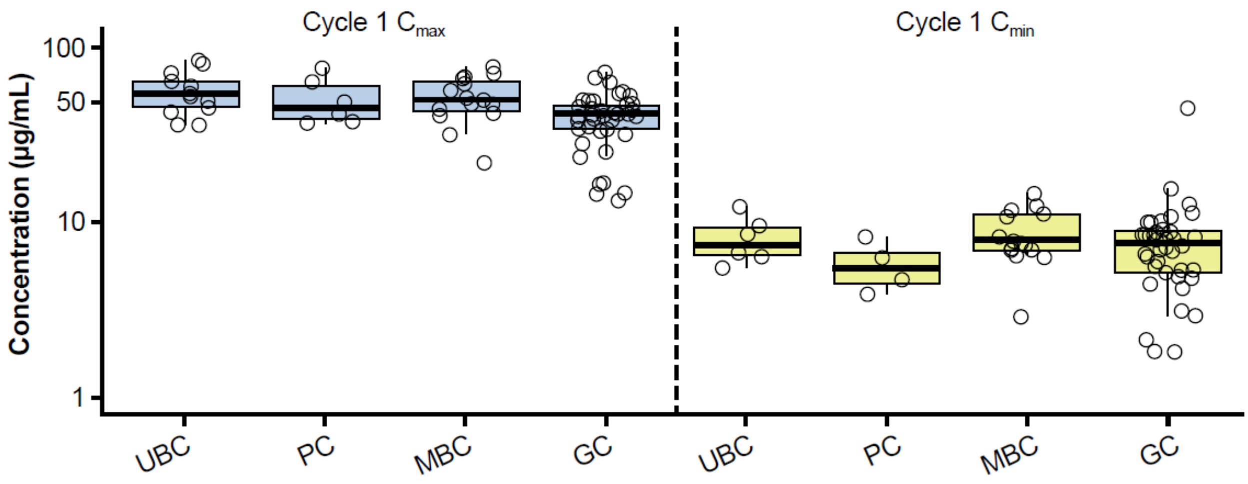

(B)

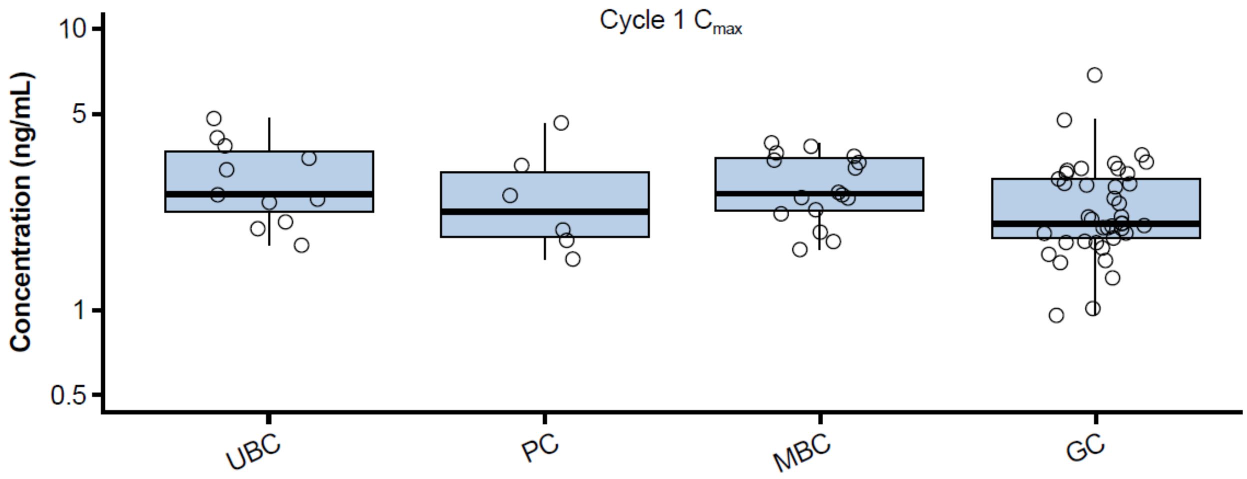

Supplemental Figure 1. Boxplots of C_{max} and C_{min} for (A) T-DM1 and (B) DM1 at Cycle 1

C_{max}, maximum concentration; C_{min}, trough concentration; DM1, emtansine; GC, gastric cancer; MBC, metastatic breast cancer; PC, pancreatic cancer; T-DM1, trastuzumab emtansine; UBC, urothelial bladder cancer.

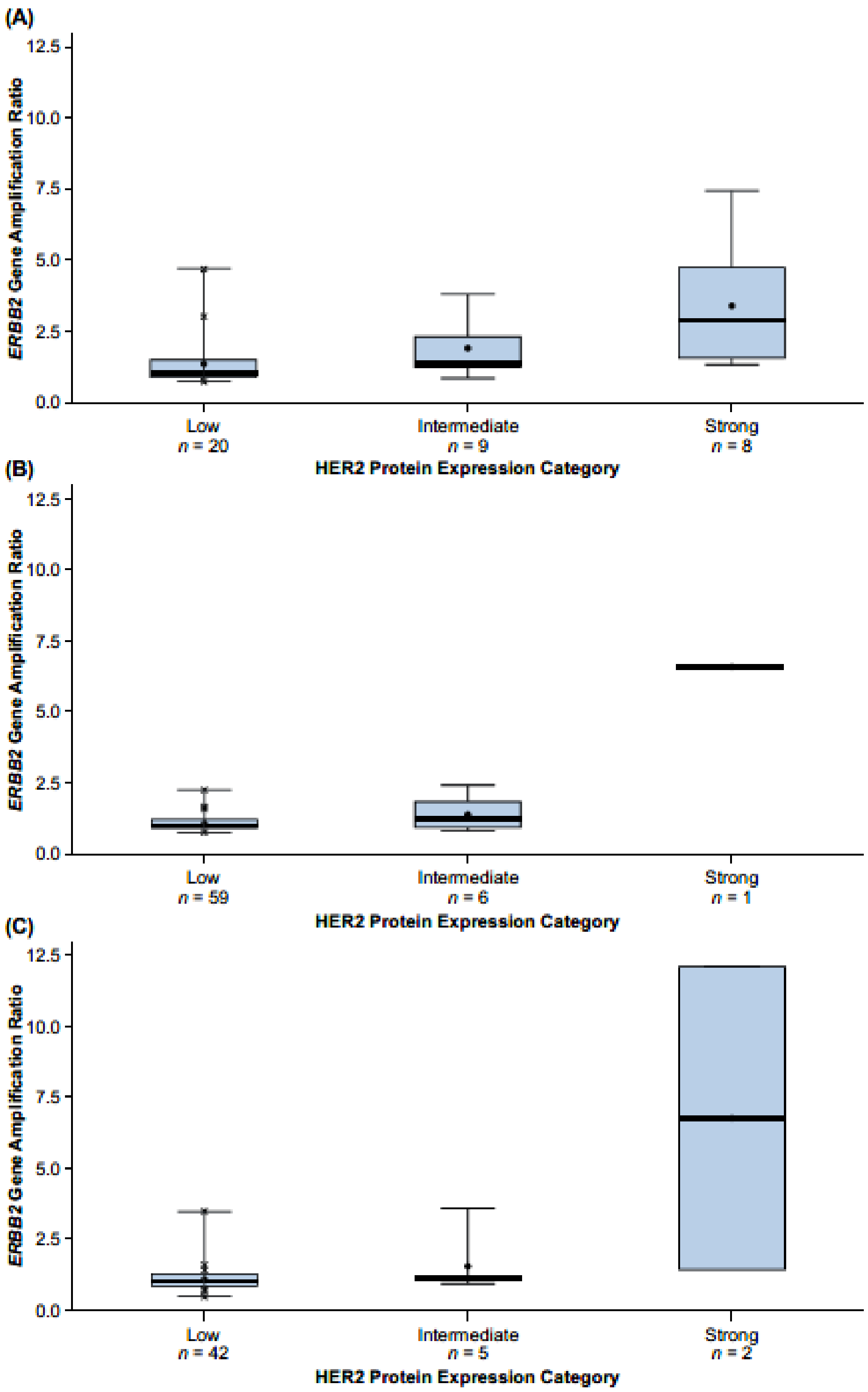

Supplemental Figure 2. HER2 protein expression category by gene amplification ratio in patients with (A) UBC, (B) pancreatic cancer, and (C) cholangiocarcinoma

ERBB2, erb-B2 receptor tyrosine kinase 2; HER2, human epidermal growth factor receptor 2; UBC, urothelial bladder cancer.

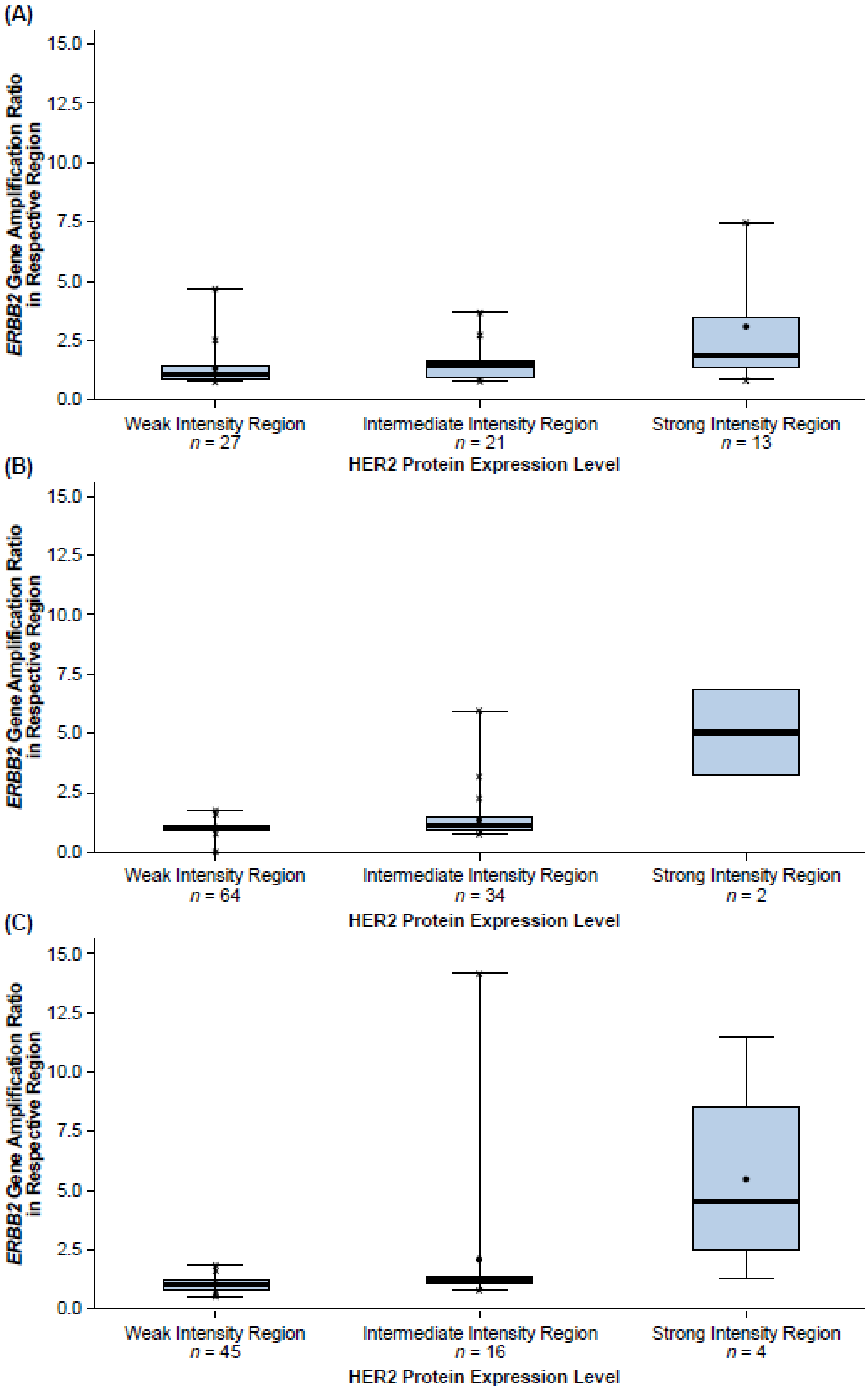

Supplemental Figure 3. HER2 expression intensity by gene amplification ratio in the respective region in patients with (A) UBC, (B) pancreatic cancer, and (C) cholangiocarcinoma

ERBB2, erb-B2 receptor tyrosine kinase 2; HER2, human epidermal growth factor receptor 2; UBC, urothelial bladder cancer.

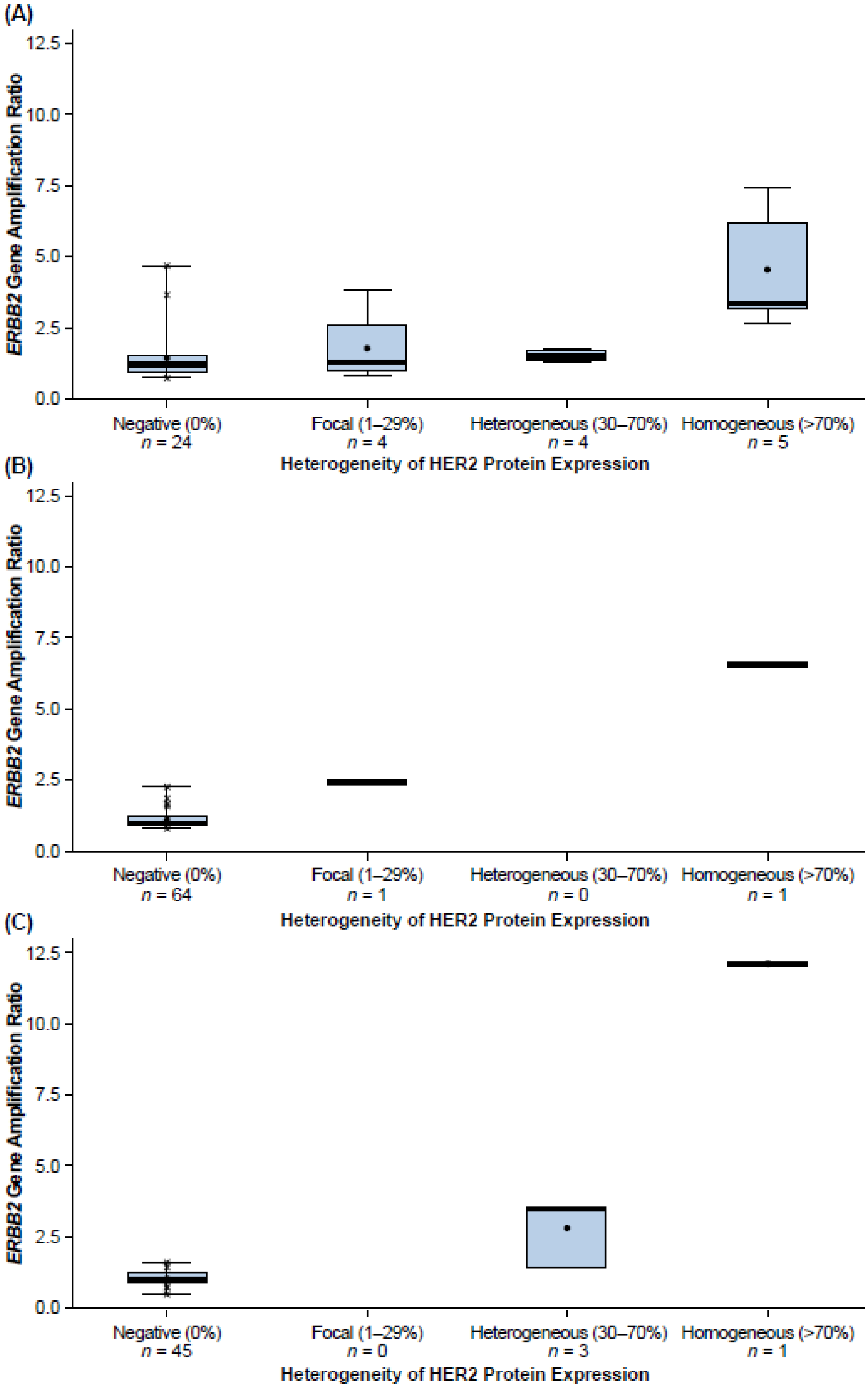

Supplemental Figure 4. Level of HER2 homogeneity by gene amplification ratio in patients with (A) UBC, (B) pancreatic cancer, and (C) cholangiocarcinoma

ERBB2, erb-B2 receptor tyrosine kinase 2; HER2, human epidermal growth factor receptor 2; UBC, urothelial bladder cancer.
